# A structural model of EFL teachers’ physical activity, emotion regulation, and competence for online teaching

**DOI:** 10.1186/s40359-024-01753-2

**Published:** 2024-05-07

**Authors:** Peng Yang Zi Cheng, Hai Liu

**Affiliations:** 1 School of Physical Education, Henan Normal University, 453000 Xinxiang, Henan Province China; 2 School of Physical Education (Main Campus), Zhengzhou University, 450001 Zhengzhou, Henan Province China

**Keywords:** ESL/EFL teachers, Mental health, Physical activity, EFL teachers, Emotion regulation, Online teaching competence

## Abstract

**Background:**

The COVID-19 pandemic has prompted a rapid shift to online teaching, placing unprecedented demands on educators’ physical and mental well-being. However, the relationship between English as a Foreign Language (EFL) teachers’ physical activity, emotion regulation, and competence for online teaching remains underexplored.

**Objectives:**

This study aimed to investigate the interplay between EFL teachers’ physical activity, emotion regulation strategies, and competence for online teaching.

**Results:**

Structural equation modeling revealed significant direct and indirect effects, indicating that physical activity positively influences emotion regulation, which, in turn, enhances teachers’ competence for online instruction. Furthermore, emotion regulation was found to mediate the relationship between physical activity and online teaching competence.

**Conclusions:**

These findings underscore the importance of promoting physical activity among EFL teachers as a means to enhance their emotion regulation skills and competence for online teaching, particularly in the context of the COVID-19 pandemic.

**Implications:**

The study highlights the need for targeted interventions aimed at supporting EFL teachers’ well-being and professional development, with implications for educational policies, teacher training programs, and institutional support structures in the digital learning landscape.

## Introduction

Physical Activity (PA) refers to any bodily movements that increase energy consumption and expenditure [1]. These activities, as described in [1], encompass a range from housework to exercise, intensive work, training and competition, and other physically demanding tasks. PA has been extensively documented to offer social, physical, and psychological benefits for adolescents. According to [2], regular engagement in PA is associated with heightened satisfaction in both school and life. Additional positive outcomes linked to PA include improved physical health in adulthood and a reduction in chronic diseases such as cancer, cardiovascular diseases, chronic headaches, and diabetes [3]. PA has also been shown to alleviate symptoms of depression, enhance academic performance, and foster high self-efficacy (SE) and positive self-image [3–5].

Conversely, inadequate engagement in physical activities is associated with poor current and future health outcomes among youth, including obesity, increased cholesterol levels, and various chronic diseases [6]. Studies have demonstrated the effectiveness of exercise and PA in promoting mental-physical health and social adjustment among the elderly [7]. Furthermore [8], highlights that PA contributes to increased happiness and self-confidence. Similarly, research conducted in Almadina City by [9] indicates a significant correlation between PA levels and the mental well-being of secondary school teachers.

Conversely, researchers have identified a lack of movement and activity as a contributing factor to reduced quality of life among older individuals [10]. However, engaging in PA can improve both mental and biological health, enhancing the overall quality of life for older adults [11]. Additionally, individuals with an active lifestyle tend to exhibit more positive psychological variables, such as reduced depression, increased satisfaction, social interaction, and trusting relationships, compared to less active or sedentary individuals. Therefore, prioritizing both PA and mental health interventions is essential to enhance the quality of life among the elderly.

Previous studies have highlighted the contribution of PA to various cognitive and affective traits. For instance, it has been stated that PA directly influences self-related functions, physical capabilities, and cognitive functions. Moreover, numerous studies have investigated the effectiveness of PA therapies in enhancing cognitive health, a crucial element for successful aging and overall well-being among the elderly [12–14]. There is a well-documented correlation between psychological well-being and PA [15], as well as between PA and the reduction of psychological distress throughout life [3, 16]. Particularly during periods of societal upheaval like the pandemic lockdowns, PA may serve as a significant contributor and predictor of psychological well-being [17]. This notion is supported by several studies conducted during the pandemic, which consistently demonstrate that a lack of PA increases psychological distress, anxiety, and depression [18].

Teachers’ well-being and mental health, which are affected by teachers’ physical activities, might contribute to several cognitive and affective variables. Among the variables that might affect teachers’ mental health and psychological well-being is emotion regulation.

Emotion regulation encompasses the intricate process of perceiving, experiencing, and expressing individual emotional changes, which can manifest both explicitly and implicitly [19]. Gross’s process model outlines four stages of emotion regulation: (1) experiencing emotions, (2) directing attention, (3) evaluating emotional responses, and (4) modulating reactions [20]. It is noted that each stage of this model is influenced by various cognitive control processes, underscoring the interconnectedness between emotions and their regulation, albeit not being synonymous. Emotion regulation is a dynamic process vulnerable to disturbances at any stage, thus emphasizing the pivotal role of enhancing emotion regulation proficiency in managing emotions effectively. Individuals who struggle with regulating their emotions often endure prolonged periods of distress, potentially culminating in clinically significant conditions such as depression or anxiety [21]. Successful emotion regulation is deemed fundamental for adaptive functioning [22], advocating for interventions aimed at bolstering emotion regulation skills to mitigate the risk of emotional disorders and foster overall well-being.

In the prevailing paradigm promoting the therapeutic benefits of exercise, considerable attention has been directed towards investigating the impact of physical activity on emotional enhancement. Notably, studies have indicated that physical exercise exerts a favorable influence on emotion regulation capacity [23], with aerobic exercise interventions demonstrating particularly pronounced effects [24]. Augustine and Hemenover (2009) similarly underscored the efficacy of aerobic exercise as an emotion regulation intervention, reporting a notable effect size of 0.47 [10]. It has been posited that moderate aerobic exercise lasting 20–30 min may yield optimal benefits for improving emotional regulation [25]. Nonetheless, some studies have yielded conflicting findings, suggesting that certain forms of physical exercise may have negligible or adverse effects on emotional regulation. For instance, investigations exploring the impact of a single session of aerobic exercise and meditation on emotional regulation failed to demonstrate significant improvements [26], while others have proposed that exceeding the ventilatory threshold during exercise may diminish pleasure and impede emotion regulation abilities [25].

**Fig. 1:**
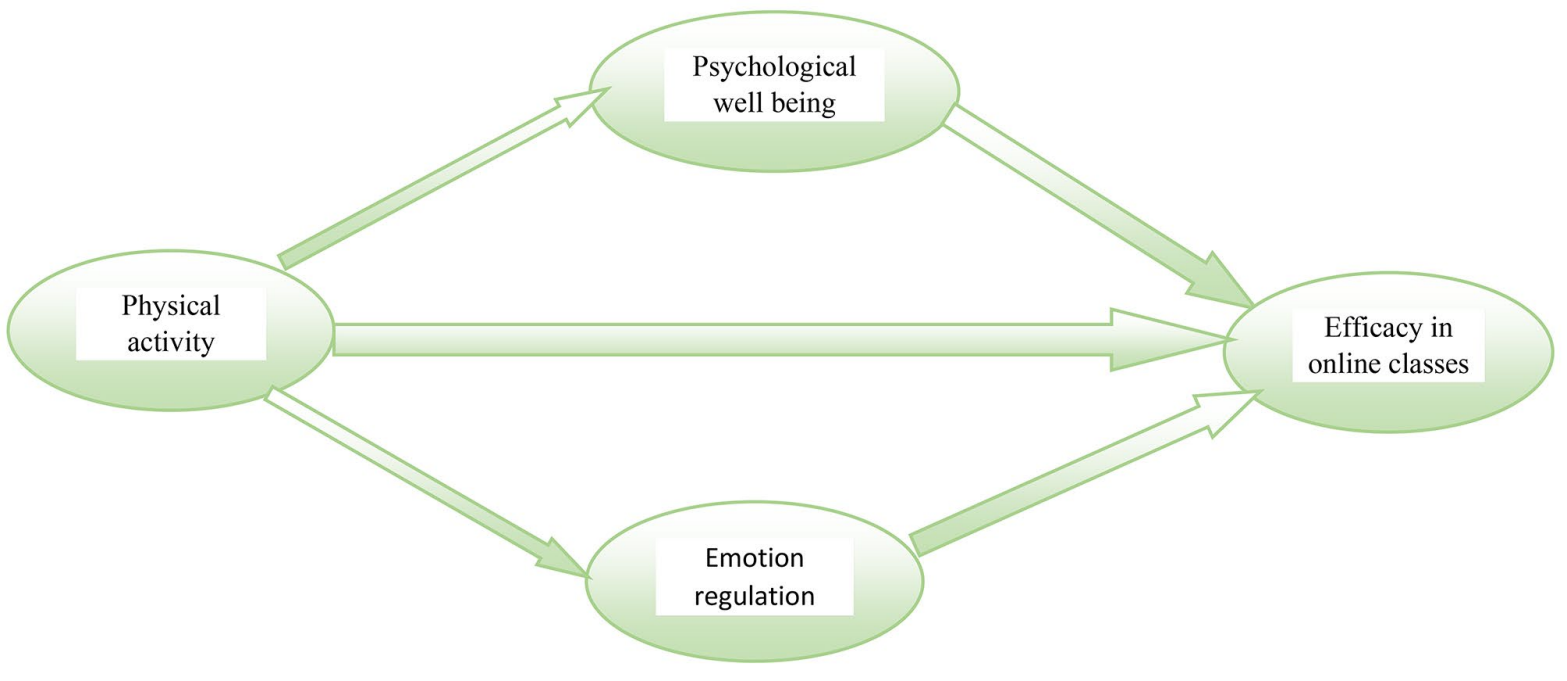
Theoretical framework of the research

The rationale for developing a structural model of EFL (English as a Foreign Language) teachers’ physical activity, emotion regulation, and competence for online teaching lies in the growing recognition of the multifaceted challenges faced by educators, particularly in the context of the COVID-19 pandemic and the transition to online instruction. While previous research has explored various factors influencing teacher effectiveness, there remains a significant gap in understanding the interplay between teachers’ physical activity, their ability to regulate emotions, and their competence in online teaching. By elucidating these relationships through a structural model, this study aims to provide valuable insights into the mechanisms underlying teacher well-being and professional performance in the digital learning environment, thereby informing targeted interventions to support educators in navigating the complexities of online instruction. The hypothetical model is presented in Fig. 1.

## Literature review

### Physical activities and mental health

The World Health Organization (WHO) demonstrated that the amount of PA for adults to have a healthy mood is assumed to be 161 min of average to vigorous activity in a week or 56 min of vigorous aerobic activity in a week. Some studies suggest moderate to intense aerobic PA during the week to be 311 min per week [26]. A meta-analysis study on adults showed that 161 min of moderate to intense PA during the week could reduce by up to 22% the risk of depression among adult men and women and create a positive self-concept among them [27]. Although most of the studies could not determine the intensity of PA, they have generally shown that a high intensity in the length of the day or week can effectively reduce depression. Other studies in the United States on adults have shown that even light PAs above 161 min per week can be effective in psychological factors such as anxiety, worry, depression, self-concept, and self-esteem [28]. Likewise [29], reported that PA is a significant cause of personal and social hygiene, and those who neglect and do not have weekly physical activities are, in fact, unaware of the significant role PA plays in human beings’ physical and mental health.

As an integral aspect of human health, mental health refers to the cognitive development process that helps people achieve their full psychological and physical potential [30]. Mental health could be associated with as satisfaction, stability, and effectiveness. It also refers to the ability of one to adapt to both their environments and others’ norms, values, and conditions happily and effectively [31].

It is also claimed that university students’ engagement in physical activities affects their mental health to a great extent. They also suggested that studying the association between the students’ physical activities and health has practical implications for the students based on which they can improve their health and quality of academic life using appropriate scientific methods. In addition [30]. PA can prevent mental disorders and problems like anxiety or depression when teachers have less or no sports or physical activities [1].

Previous studies have shown that the amount of light PA effectively reduces depression and anxiety. For this reason, physically inactive people have the highest risk of depression and low self-esteem [32]. Therefore, researchers have recommended having light or moderate PA during the week to prevent mental health risks. Moreover, recent studies have shown that the intensity of PA is effective in psychological factors. However, the duration of PA is also effective, so an amount of 11 to 16 min of activity is adequate. PA during the day can reduce depression, anxiety, and worry among people, leading to high self-esteem and positive physical self-concept [33].

Although Costigan and colleagues state that interval training with high intensity leads to increased physical and mental health among teenagers, some studies reject how high-intensity interval training works in lowering the depression and increasing SE and physical self-concept. Therefore, concerning the duration and amount of PA, it cannot be definitively stated that any type of PA can be effective in all psychological factors of adults.In their research [3], showed that PA increases happiness and self-confidence, reduces depression and anxiety, and increases physical and mental health throughout life. Researchers have mentioned that lack of movement and activity is the reason for the low quality of life of the elderly [34], stated that participating in short time PA also increases physical and mental health and life quality in the elderly. This is critical to ensuring that elderly continue to enjoy a high quality of life into their golden years.

In another study [35], examined AJA employees’ PA and its relationship to psychological factors (physical self-concept, mental resilience, depression and anxiety). They found that the PA level of the AJA employees was acceptable. The PA significantly correlated with workers’ personalities and temperaments [1]. showed that indoor PA is preventive in lockdown situations, while activity level does not affect mental health. It has been claimed that PA improves students’ interpersonal skills, intelligence level, and emotional and psychological state [36].

### Teachers’ PA and their well-being

Psychological well-being is a multidimensional structure that includes individual and social aspects that affect how we perceive the world in which we live and how we regulate our behaviour while facing problems and challenges. Psychological well-being highly depends on one’s ability to regulate emotions [37]. In recent years, some authors have suggested that engaging in PA is one of the ways to facilitate psychological well-being [36]. PA and sports are essential aspects of human health because, in addition to promoting metabolic activity and cardiovascular functions, it is a crucial factor contributing to a general sensation of psychological well-being [37]. Over the past two decades, researchers have focused on the connectionbetween psychological well-being and PA. Some other researchers in different contexts have confirmed the positive association between people’s well-being and physical activities [16, 38].

PA is associated with an appropriate quality of life, life satisfaction, and happiness and provides people with resources and structures to enjoy life as reported by previous studies. For example [39], has maintained that doing physical activities through independent activities or regular and frequent sports programs fosters well-being, mental health, flexibility, memory, autonomy, optimism, body image, and emotional clarity.Overall, it has been observed that PA and sport are correlated with well-being based on the discovery of [40] carried out in 10 European countries with over eleven thousand young people. They also found positive correlations between the frequency of PA and well-being in both male and female adolescents.

Similarly [41], discovered that playing sports has benefits, including the promotion of values such as teamwork, collaboration, and autonomy development, which in turn aid in the acquisition of improved life skills [42]. recently reported that regular exercise and physical activities significantly impact their self-esteem. The study by [43] showed that suitable sports programs could effectively improve women’s self-esteem and satisfaction in life and mood during the quarantine period of the Covid-19 virus. Doing light PA when covid-19 pandemic will ease the adverse mental health effects of social distancing [44] [37]. have argued that engaging in exercise and PA notably impacts various dimensions of psychological well-being, specifically self-acceptance, purpose in life, and positive relations with others. Also [45], indicated that people with more PA during quarantine restrictions reported less anxiety than those with less PA during quarantine.

In |Iran context, the correlation between women’s PA and their psychological well-being was investigated [45]. A positive and statistically significant correlation is found between the level of psychological well-being and PA(*P* = .643). According to the results, PA in the post-coronary period can help reduce the adverse psychological effects of social distancing and adherence to quarantine while coronation period [46]. conducted a study using pre-test/post-test experimental research design. Their study findings revealed that PA significantly enhanced the participants’ mental and psychological well-being. Moreover [47], investigated the effect of a surfing program on a group of children/young people. They concluded that engagement in sports and physical activities increased the participants’ satisfaction with appearance and changed their attitudes towards schools.

### Teachers’ emotion regulations

Emotion regulation, the dynamic process of managing and modulating emotional experiences, plays a pivotal role in both personal and professional well-being [48]. Individuals utilize various strategies, such as situation selection, attentional deployment, or cognitive change, to navigate their emotions and adapt to diverse demands [49, 50].

Within organizational settings, emotion regulation becomes particularly relevant as employees strive to balance authentic emotional expression with professional expectations [51]. Research suggests the strategic use of regulation strategies, like deep acting (aligning internal feelings with expected emotions) or surface acting (displaying emotions without genuinely feeling them), to conform to organizational norms [52, 53].

The educational sector presents a unique environment where emotion regulation, often synonymous with emotional labor, takes center stage [54]. Teachers constantly engage in emotion regulation during interactions with students, colleagues, and parents, aiming to cultivate a positive and conducive learning environment [55, 56]. Research consistently emphasizes the emotional demands placed on teachers, highlighting the crucial role of emotion regulation in effectively managing these inherent stressors [57]. Beyond professional requirements, emotion regulation becomes an integral aspect of fostering effective teaching and learning experiences for educators. The strategies employed by teachers influence not only their own well-being but also the emotional climate of the classroom and, ultimately, student outcomes [58].

A growing body of research points towards a significant association between teachers’ emotion regulation abilities and their susceptibility to burnout. For instance, Brackett et al. [59] found that British secondary school teachers with stronger emotion regulation skills reported higher job satisfaction and lower burnout. Similarly, Ghanizadeh & Royaei [60] concluded that effective emotional regulation strategies played a crucial role in mitigating burnout among language teachers. Chang’s [61] theoretical model emphasizes the cognitive processes involved in managing emotions, suggesting that teachers’ ability to appraise, regulate, and cope with student misbehavior significantly impacts their emotional well-being and burnout.

Further studies by Fathi et al. [62] and Bing et al. [63] explored this link in the context of EFL teachers. Both studies identified stronger self-efficacy (confidence in one’s abilities) and effective emotion regulation skills as protective factors against burnout, highlighting the potential benefits of interventions that enhance both aspects.

### Teachers’ competecnce for online teaching

Numerous competencies are acknowledged as exemplary practices for instructors in online settings, as evidenced by scholarly research [64, 65]. However, closer examination of these identified competencies has revealed discrepancies, particularly in models tailored specifically for online teaching environments. Baran et al. [66] observed that the essential roles and competencies expected from online instructors often vary in the literature, depending on the particular context of online instruction. Consequently, the dynamic nature of the online learning environment necessitates educators to possess a diverse range of competencies.

In a comprehensive literature review, Thomas and Graham [67] emphasized that previous studies had assessed various competencies for online teaching, with course design emerging as the most extensively researched area. In contrast, Bigatel et al. [68] outlined competencies for online teaching that focused primarily on teaching behaviors. They delineated tasks related to extensive instructional elaboration, involving evaluators, course developers, online instructors, and academic staff, encompassing a total of 64 online teaching behaviors. The investigation involved 197 participants, who utilized a 7-point Likert scale to indicate their level of agreement with each assessment, identifying tasks perceived as most crucial in online teaching contexts. Through exploratory factor analysis, the researchers grouped the tasks into seven competency domains: (1) administration/leadership, (2) active learning, (3) multimedia technology, (4) active teaching/responsiveness, (5) technological competence, (6) policy enforcement, and (7) classroom management. Bigatel et al. [67] introduced a model elucidating educators’ teaching behaviors during course delivery, which did not prioritize the factor of course design. This model serves as the basis for the present study. While acknowledging potential limitations, further validation checks or suggestions for enhancement are imperative to evaluate its accuracy.

### Development of the hypotheses

Congruent with the proposed theoretical framework and the previous findings, the following hypotheses were speculated.

H1 = Teachers’ physical activities affect their mental health.

H2 = EFL Teachers’ physical activities affect emotion regulation.

H3 = EFL Teachers’ physical activities affect their competence for online teaching.

H4 = EFL teachers’ mental health has a significant direct impact on competence for online teaching.

H5 = EFL teachers’ emotion regulation has a significant direct impact on their competence for online teaching.

H6 = Physical activities significantly affects teachers’ competence for online teaching through mental health.

H7 = PA significantly affects teachers’ competence for online teaching through emotion regulation .

## Methodology

### Participants

The participants wee selected from Xinxiang and Zhengzhou in china, revise the text based on this revision in the setting: The participate invitation was sent to 400 English teachers in different. Only 364 teachers returned the survey to the researchers. The study included a sample of full-time EFL teachers who were purposefully recruited from a diverse range of schools and language institutes located in different provinces, cities, and areas in Xinxiang (*n* = 190) and Zhengzhou (*n* = 174) through convenience sampling. Table 1 summarizes the participants’ demographic information. Based on Table 1, the participants male were 170 (47.8%) and female were 194 (52.2%) full-time teachers were selected. To have a homogenous sample, the researchers avoided including part-time teachers. The participants’ ages spanned from 23 to 54 years old (M = 26.7, SD = 8.1), Additionally, theirlecturing experience were ranged from 3 to 30 years (M = 12.1, SD = 4). Another inclusion criterion was the teachers’ majors. Only teachers with English language majors from colleges and teacher education centreswere recruited. The participants knew the researchers’ intents and objectives and completed and signed the informed consent forms. Respondents were assured that their responses would be subjected to anonymous analysis and would not have any detrimental or beneficial impact on their employment situation.

**Table 1:** Participants’ demographic characteristics

Variable			Number (%)
Region	Xinxiang		174 (47.8)
	Zhengzhou		190 (52.2)
Gender	Male		170 (46.7)
	Female		194 (53.3)
Age	23–30		110 (30.21)
	30–45		200 (54.9)
	45–54		54 (15)
Teaching experience		1–10	125 (34.3)
		10–20	140 ( 38.5)
		20–30	99 (27.2)

### Research instruments

The researchers utilised four established instruments to gather the required information to fulfil the study’s objectives.

#### Short Form Questionnaire of International Physical Activities (SE-IPAQ)

The present questionnaires consist of a scale of seven items that assess self-reported physical activities. This scale was originally established by [68]aims to quantify the extent of vigorous- and moderate-intensity PA, as well as sitting and walking, engaged in by respondents during the preceding seven-day period [68]. As an illustration, the respondents were inquired about the frequency of engaging in intense activities such as exercise classes, weightlifting, or rapid cycling for at least 10 min per day throughout the previous week. The participants were instructed to report the duration of vigorous PA they engaged in during any one of the preceding seven days. The present study’s questionnaire exhibited strong internal consistency and validity, as evidenced by previous study conducted by [69]. In order to assess the frequency component, we assigned a value of 0 to activities of the indeterminate event, 1 to activities occurring once per week, 2.5 to activities occurring 2–3 times per week, 5 to activities occurring 4–6 times per week, and 7 to activities occurring every day of the week. The PA score exhibited a range spanning from 0 to 7.

### Emotion Regulation Questionnaire (ERQ)

The Emotion Regulation Questionnaire (ERQ) was utilized in this study to measure the participants’ ability to regulate their emotions. Developed by Gross and John [70], this scale assesses two main categories of emotion regulation strategies: cognitive reappraisal (CR) and expressive suppression (ES). Reappraisal involves changing the way a situation is perceived to reduce its emotional impact, while suppression involves inhibiting the outward expression of inner feelings. The scale comprises six items for cognitive reappraisal, with an example item being “When I’m faced with a stressful situation, I make myself think about it in a way that helps me stay calm” and four items for expressive suppression, with an example item being “When I am feeling positive emotions, I am careful not to express them.” Participants rated their responses on a seven-point Likert scale ranging from 1 (“strongly disagree”) to 7 (“strongly agree”).

### Mental health scale

To assess the mental well-being of the participants, the general mental health questionnaire-28, formulated by [71]was applied. Several researchers in different contexts further localized this questionnaire developed by [72]. This 28-item scale consists of four dimensions which measure the participants’ public health (anxiety, somatic symptoms, and insomnia, social dysfunction, and depression). Each dimension has 7 items, measured on a Likert scale.

Online teaching competence scale.

The Online Teaching Competence scale, is a 30-item scale employed to evaluate online teaching competencies [68]. Sample items included assessing instructors’ encouragement of student interaction through team tasks and projects and monitoring adherence to academic integrity policies. The scale demonstrated good internal consistency, with a calculated Cronbach’s alpha of 0.83.

### Procedure

To fulfill the study’s objectives, we conducted a web-based survey targeting EFL instructors across primary and secondary schools, as well as universities, in northern China. We leveraged the WeChat platform to invite potential participants. The survey’s introductory page provided a straightforward overview of the research aims and explicitly solicited informed consent from users. We emphasized that any personal data and responses would remain strictly confidential and secured within a password-protected system at the research center, solely for research purposes. To bolster the online survey’s trustworthiness and validity, the research team included contact details (email and WeChat ID) for participants encountering technical difficulties or seeking clarification about the survey.

We implemented several safeguards to ensure data quality and reliability. First, the online survey underwent meticulous design and testing before distribution. Second, the survey link was delivered directly to participants’ individual WeChat accounts, and our contact information was provided to address any inquiries or challenges with the survey completion. Third, clear instructions were provided on how to complete the survey, and participants were required to confirm informed consent before proceeding. Additionally, we prioritized participant privacy and confidentiality by refraining from collecting identifiable information such as names, addresses, or phone numbers. Finally, we adhered to all ethical considerations throughout the entire research process, including acquiring ethical approval from our institution’s relevant committee.

### Data analysis

The study’s data underwent analysis through SPSS (Version 26) and Amos (Version 26) using Maximum Likelihood Estimation (MLE). The researchers thoroughly checked the data for outliers, missing data, and multivariate normality. Results indicated a low rate of missing data (ranging from 0.6 to 1.5%), which met the requirements for missing data to be completely at random (MCAR) based on Little’s test (χ2 = 1053.26, *p* = .478). To impute the missing data, Expectation Maximization (EM) was used, a robust estimation method for SEM [65]. Univariate outliers were detected through scatter plots and Z-standardized values that should fall between − 3 and + 3, leading to the removal of eight cases. Additionally, normality was not violated as the skewness and kurtosis values fell within the acceptable range of − 1 and + 1 (see Table 2). Meyers et al.’s [66] recommendation of using Mahalanobis distances to identify multivariate outliers was utilized, and one case was removed because its value exceeded the critical chi-square value of 0.001. The study’s final sample size was 377 participants, with a gender distribution of 44% male (*n* = 166) and 56% female (*n* = 211).

In addition, to ensure the appropriateness of the chosen scales for this specific context, we conducted confirmatory factor analyses (CFAs). The results, presented in Table 2, confirmed an excellent fit for the models, indicating the scales accurately captured the intended constructs. Subsequently, structural equation modeling (SEM) was employed to investigate the mediating effect of emotional labor on the relationship between teacher emotion regulation and teacher well-being.

## Results

In the present investigation, the study scrutinized the means and standard deviations of the three variables in male and female instructors. The average scores and standard deviations of burnout for males and females were 3.75 ± 0.81 and 3.84 ± 0.71, respectively. Regarding teacher emotion regulation, males scored an average of 3.28 ± 0.72 while females scored an average of 3.11 ± 0.81. For emotional labour, the means were 4.03 ± 0.89 for males and 4.16 ± 0.92 for females. After conducting independent samples t-tests, no significant differences were found between males and females in burnout (t = -0.932, *p* = .352), teacher emotion regulation (t = 0.986, *p* = .326), and emotional labour (t = -1.244, *p* = .216). Results are presented in Table 2.

**Table 2:** CFA results

	CMIN	DF	CMIN/DF	*P*	CFI	RMSEA	SRMR	α
Physical Activities	195.32	100	1.953	< 0.001	0.952	0.061	0.046	0.77
Emotion regulation	94.276	49	1.924	< 0.001	0.978	0.042	0.036	0.89
Mental health	138.05	74	1.865	< 0.001	0.981	0.039	0.032	0.88
Online teaching competence	130.05	74	1.865	< 0.001	0.981	0.039	0.032	0.88

### Validity and reliability analysis

Table 2 presents the results of first-order confirmatory factor analyses (CFAs) and the reliability indices of the three measurement scales: Burnout, Emotion Regulation, and Emotional Labour. The CFAs were conducted using the Maximum Likelihood Estimation method. The results of the CFAs show that all three measurement models have good fit based on the Hu and Bentler’s guidelines. The CMIN values for Burnout, Emotion Regulation, and Emotional Labour are 195.328, 94.276, and 138.054, respectively, with corresponding degrees of freedom of 100, 49, and 74. The CMIN/DF ratios are less than 2, indicating good model fit. The *P*-values are all less than 0.001, indicating that the models fit the data well. The CFI values are 0.952, 0.978, and 0.981 for Burnout, Emotion Regulation, and Emotional Labour, respectively, which suggest good model fit. The RMSEA values are 0.061, 0.042, and 0.039 for Burnout, Emotion Regulation, and Emotional Labour, respectively, which also indicate good model fit. The SRMR values are 0.046, 0.036, and 0.032 for Burnout, Emotion Regulation, and Emotional Labour, respectively, which are within acceptable ranges. Finally, the reliability indices (Cronbach’s alpha) for Burnout, Emotion Regulation, and Emotional Labour are 0.77, 0.89, and 0.88, respectively, indicating good internal consistency (See Table 3).

**Table 3:** Descriptive statistics and correlations between the constructs

Constructs	1	2	3	4
1. physical activity	1			
2. mental health	0.51**	1		
3. emotion regulation	0.42**	0.46**	1	
4.Online teaching competence	0.53	0.49	0.43	1
4. Mean	3.58	3.69	3.23	3.52
5. SD	0.78	0.81	0.77	0.81
6. Skewedness	−0.36	−0.21	0.16	0.15
7. Kurtosis	−0.51	−0.81	−0.48	− 0.23

Note. * *p* < .05 ** *p* < .01.

The study examined the interrelationships between four key constructs: physical activity, mental health, emotion regulation, and online teaching competence. Results revealed significant correlations among these constructs, shedding light on their interconnected nature. Firstly, physical activity exhibited a positive correlation with both mental health (*r* = .51, *p* < .01) and emotion regulation (*r* = .42, *p* < .01). This suggests that engaging in regular physical activity is associated with better mental health and improved ability to regulate emotions.

Similarly, mental health demonstrated a positive correlation with emotion regulation (*r* = .46, *p* < .01), indicating that individuals with better mental health tend to exhibit more effective emotion regulation strategies. Moreover, online teaching competence displayed positive correlations with physical activity (*r* = .53), mental health (*r* = .49), and emotion regulation (*r* = .43). This implies that higher levels of online teaching competence are linked with increased engagement in physical activity, enhanced mental well-being, and more proficient emotion regulation skills. In terms of mean scores, mental health emerged as the highest-rated construct (Mean = 3.69), followed closely by physical activity (Mean = 3.58), online teaching competence (Mean = 3.52), and emotion regulation (Mean = 3.23). Additionally, the standard deviations indicated variability within each construct, with mental health and physical activity showing slightly higher variability compared to emotion regulation and online teaching competence. Regarding the distribution characteristics, all constructs displayed slightly negative skewness and platykurtic distributions, suggesting a concentration of data towards the higher end of the scale and fewer extreme values. Overall, these findings underscore the intricate connections between physical activity, mental health, emotion regulation, and online teaching competence, highlighting the importance of considering these factors collectively in understanding individuals’ well-being and professional competence in online teaching settings.

### SEM analysis

After conducting SEM to investigate the mediating role of emotional labor in the relationship between emotion regulation and teacher burnout, the goodness of fit for the proposed structural model was assessed using various fit indices for both male and female groups. The findings revealed that the suggested model had a satisfactory fit with the data for males (χ²/df = 1.490, CFI = 0.942, TLI = 0.937, IFI = 0.942, RMSEA = 0.042, and SRMR = 0.056) and females (χ²/df = 1.573, CFI = 0.940, TLI = 0.935, IFI = 0.938, RMSEA = 0.044, and SRMR = 0.057). The standardized parameter estimates for the proposed model are displayed in Fig. 1. Furthermore, a multi-group invariance analysis was performed to examine whether the model coefficients differed significantly between genders. The results of the χ² difference test between constrained and unconstrained models (Δχ² = 5.551, Δdf = 5, *p* = .361) revealed that the model coefficients in the suggested mediation model were equivalent across male and female groups. Therefore, there were no notable disparities between male and female teachers regarding both direct and indirect effects of the predictor variable on the outcome variable. Additionally, bootstrap resampling was utilized with 500 iterations to assess the sampling distribution and evaluate the indirect effects in both male and female groups.

The findings from the first research question indicated that teacher emotion regulation had a significant and positive correlation with both emotional labour (β = 0.542) and burnout (β = 0.334), whereas emotional labour had a positive association with burnout as well (β = 0.478). As for the second research question, emotional labour was identified as a mediator in the relationship between teacher emotion regulation and burnout, with an indirect effect of β = 0.259. Together, teacher emotion regulation and emotional labour accounted for 48.26% of the variance in burnout, with the remaining variance explained by external factors. Table 4 illustrates the direct and indirect effects of the structural model, along with the 95% confidence intervals and the magnitude-of-effect estimates (*f*2) to assess the effect sizes (See Table 4).

**Table 4:** Evaluation of the hypotheses

Model pathways	B	SE	Β	*P*
*Direct effects*				
Physical activities $\rightarrow$ mental health	0.502	0.162	0.392	< 0.001
Physical activities $\rightarrow$ emotion regulation	0.762	0.183	0.536	< 0.001
Physical activities $\rightarrow$ online teaching competence	0.52	0.125	0.493	< 0.001
Emotion regulation $\rightarrow$ online teaching competence	0.48	0.113	0.46	< 0.001
Mental health $\rightarrow$ online teaching competence	0.51	0.118	0.50	< 0.001
*Indirect effect*				
Physical activities $\rightarrow$ mental health $\rightarrow$ online teaching competence	0.408	0.092	0.264	< 0.001
Physical activities $\rightarrow$ mental health $\rightarrow$ online teaching competence	0.420	0.096	0.268	< 0.001

As seen in Table 4, the model pathways analysis uncovered significant direct and indirect effects among the variables, shedding light on the intricate relationships between physical activities, mental health, emotion regulation, and online teaching competence. Engaging in physical activities demonstrated positive direct effects on mental health (β = 0.392, *p* < .001), emotion regulation (β = 0.536, *p* < .001), and online teaching competence (β = 0.493, *p* < .001), indicating that individuals who participate in physical activities tend to exhibit better mental health, improved emotion regulation skills, and enhanced online teaching abilities. Additionally, emotion regulation exhibited a significant direct effect on online teaching competence (β = 0.46, *p* < .001), suggesting that individuals with effective emotion regulation skills are more likely to demonstrate higher levels of competence in online teaching. Similarly, better mental health was found to be positively associated with online teaching competence (β = 0.50, *p* < .001), highlighting the importance of mental well-being in facilitating effective online teaching practices.

Moreover, the analysis revealed significant indirect effects, particularly through the pathway of physical activities to mental health to online teaching competence. The indirect effect of physical activities on online teaching competence through mental health was β = 0.264 (*p* < .001), while the indirect effect through emotion regulation was β = 0.268 (*p* < .001). This suggests that engaging in physical activities indirectly contributes to online teaching competence by positively influencing mental health and emotion regulation, which in turn enhance educators’ abilities in online teaching environments. These findings underscore the interconnected nature of physical activities, mental health, emotion regulation, and online teaching competence, emphasizing the importance of holistic approaches to teacher training and support, particularly in the context of online education.

## Discussion

### H1: physical activities → mental health

The hypothesis posits that physical activities have a positive association with mental health. This conjecture is supported by a plethora of studies across diverse populations. For instance, Aperribai et al. found that engaging in physical activity during the COVID-19 lockdown was linked to improved mental well-being among teachers [1]. Similarly, Vindegaard and Benros conducted a systematic review highlighting the beneficial effects of physical activity on mental health, particularly during times of stress such as the pandemic [38]. Furthermore, research by Ai et al. underscores the importance of physical exercise in mitigating mental health challenges, suggesting a robust link between physical activities and positive mental health outcomes [29].

### H2 = physical activities $\rightarrow$ emotion regulation

This hypothesis proposes that engaging in physical activities positively influences emotion regulation abilities. Evidence supporting this notion comes from various sources. Gross and John demonstrated that regular physical activity is associated with enhanced emotion regulation processes, leading to improved overall well-being [70]. Additionally, research by Bernstein and McNally found that acute aerobic exercise helps individuals overcome emotion regulation deficits, suggesting a direct link between physical activities and effective emotion regulation strategies [23]. Moreover, Edwards et al. conducted a randomized controlled intervention study showing that acute exercise can lead to improvements in emotional regulation, further strengthening the hypothesis [24].

### H3 = physical activities $\rightarrow$ online teaching competence

The hypothesis suggests that physical activities positively correlate with online teaching competence. This assertion is supported by literature emphasizing the cognitive benefits of physical exercise. For instance, Schuch et al. conducted a meta-analysis of prospective cohort studies and concluded that physical activity protects against incident anxiety, which may contribute to better cognitive function and job performance, including online teaching competence [27]. Furthermore, research by Albrahim focused on online teaching skills and competencies, highlighting the importance of maintaining physical well-being to support effective online instructional practices [64].

### H4 = emotion regulation $\rightarrow$ online teaching competence

This hypothesis proposes a positive relationship between emotion regulation abilities and online teaching competence. Existing literature provides substantial support for this assertion. Brotheridge and Lee highlighted the critical role of emotion regulation in managing the demands of teaching, suggesting that effective regulation strategies are essential for professional efficacy [49]. Additionally, Sutton and Mudrey-Camino emphasized the importance of emotion regulation in classroom management, which directly translates to online teaching settings [57]. Moreover, Keller et al. conducted an experience sampling study demonstrating that teachers’ emotional experiences and regulation strategies significantly influence their classroom performance, providing further credence to the hypothesis [63].

### H5 = mental health $\rightarrow$ online teaching competence

This hypothesis suggests that mental health positively influences online teaching competence. Research in this area indicates a strong connection between mental health and job performance. For example, McMahon et al. conducted a study examining the impact of mental health on work-related outcomes and found that employees’ mental health significantly predicted their job performance [40]. Similarly, Fathi et al. explored the relationship between self-efficacy, reflection, burnout, and mental health among EFL teachers, revealing that mental health factors play a crucial role in determining teaching effectiveness [61]. These findings lend support to the hypothesis that better mental health contributes to enhanced online teaching competence.

### H6 = indirect effect: physical activities $\rightarrow$ mental health $\rightarrow$ online teaching competence

This hypothesis proposes an indirect pathway through which physical activities influence online teaching competence via the mediation of mental health. The literature provides compelling evidence for this indirect effect. Every-Palmer et al. conducted a cross-sectional study during the COVID-19 lockdown and found that psychological distress and anxiety significantly impacted individuals’ well-being, including their professional performance []. Moreover, Northey et al. conducted a systematic review with meta-analysis, revealing that exercise interventions positively affect cognitive function and mental well-being, which may ultimately enhance job performance, including online teaching competence [15].

### H7 = indirect effect: physical activities $\rightarrow$ emotion regulation $\rightarrow$ online teaching competence

Finally, this hypothesis suggests that physical activities influence online teaching competence indirectly through the mediation of emotion regulation abilities. Research supports this pathway, highlighting the interconnectedness of physical activities, emotion regulation, and professional competence. Edwards et al. demonstrated that acute exercise leads to improvements in emotion regulation, which in turn may enhance job performance [24]. Additionally, Bernstein and McNally found that acute aerobic exercise helps individuals overcome emotion regulation deficits, suggesting that physical activities play a crucial role in fostering effective emotion regulation strategies that are essential for successful teaching practices, including online instruction [23]. Moreover, Sutton and Mudrey-Camino emphasized the importance of emotion regulation in classroom management, which directly translates to online teaching settings [57].

## Conclusions and implications

The synthesis of literature underscores the indispensable role of physical activities in fostering both the mental health and emotion regulation capacities of educators, especially in the context of challenges such as the COVID-19 pandemic. The reviewed studies consistently highlight the positive correlation between regular physical exercise and enhanced well-being, indicating that engaging in physical activities contributes significantly to mitigating stress and promoting psychological resilience among teachers. Moreover, the research underscores the critical link between physical activities and emotion regulation, suggesting that habitual exercise fosters effective coping mechanisms and emotional stability, which are invaluable assets for educators navigating the complexities of online teaching.

These findings carry profound implications for educational institutions and policymakers seeking to support the holistic well-being and professional efficacy of teachers. Prioritizing initiatives that promote physical activities and mental health awareness among educators can yield substantial benefits, not only for individual teachers but also for the overall educational ecosystem. By investing in teacher training programs that incorporate stress management techniques, emotion regulation strategies, and wellness practices, institutions can empower educators to navigate the demands of online instruction with resilience and effectiveness. Furthermore, fostering a culture of holistic well-being within educational settings, characterized by institutional support, resources for self-care, and a nurturing work environment, holds the potential to enhance teacher satisfaction, retention, and ultimately, student success. Continued research into the specific mechanisms underlying the relationship between physical activities, mental health, and teaching competence will further inform evidence-based interventions aimed at promoting educator well-being and advancing educational outcomes.

### Limitations

Despite the merits of this study, we need some help with collecting and analyzing the data. First, we used the short form of IPAQ, a self-report assessment. The data would have been richer if the long-form IPAQ had been used and other techniques such as observations and interviews had been used. The contribution of the PA to the sub-components of mental health, well-being, and SE, because the journals’ word limits were not included in the analysis and discussion sections. Another area for improvement is the researchers’ inability to recruit participants from one country to have a homogenous group of respondents. In this study, the structural relations among the research constructs were investigated. It is suggested that further researchers explore the interdependent relationships between the constructs under study and the constituent elements of each construct. The teaching experience, nationality, and gender of the participants may also moderate the correlations among the constructs and their effects on each other. Further studies are recommended to replicate the study using large-scale data focusing on the abovementioned limitations.

## Data Availability

The datasets used and/or analysed during the current study available from the corresponding author on reasonable request.
